# Mycotic Aortic Aneurysm Secondary to Salmonella enterica Infection: A Case Report and Treatment Approach

**DOI:** 10.7759/cureus.56399

**Published:** 2024-03-18

**Authors:** Samah A Elshweikh, Amr Abdellatif Ibrahim, Waleed Saleh Almutairi, Fahad AlHarbi, Abdullah A Alrasheedi, Ahmed Negm Eldine Said Mubark, Eman Ibrahim Basha, Reham M Elkolaly

**Affiliations:** 1 Internal Medicine/Hematology, Tanta University, Tanta, EGY; 2 Vascular Surgery, Zagazig University, Zagazig, EGY; 3 Radiology, Buraidah Central Hospital, Qassim, SAU; 4 Pulmonology, Buraidah Central Hospital, Qassim, SAU; 5 Infectious Diseases, Buraidah Central Hospital, Qassim, SAU; 6 Clinical Pathology, Buraidah Central Hospital, Qassim, SAU; 7 Chest Diseases, Faculty of Medicine, Tanta University, Tanta, EGY

**Keywords:** endovascular aortic repair, endovascular infection, bacteremia, salmonella enterica, mycotic aortic aneurysm

## Abstract

Mycotic (infected) aortic aneurysm is a severe clinical condition with high morbidity and mortality. *Salmonella *spp. is a Gram-negative, rod-shaped bacteria that is typically limited to the gastrointestinal tract and resolves spontaneously but can progress to invasive infections such as bacteremia. Serious complications may arise, particularly in debilitated, elderly, and neonatal patients.

We describe the case of a 74-year-old female with a history of diabetes and hypertension who presented with shortness of breath, fever, chills, abdominal pain, vomiting, and diarrhea. The patient’s blood culture tested positive for *Salmonella enterica*, and she was given ceftriaxone based on the results, but he remained symptomatic. A computed tomography scan of the chest with contrast revealed a mycotic aneurysm of the thoracic aorta.

The patient was urgently transferred to a higher level of care and underwent emergency thoracic endovascular aortic repair with stenting and intravenous antibiotics. The presence of an infected aneurysm and associated abscess formation in such high-risk patients makes the endovascular approach more suitable than other options such as open surgery, aneurysmal excision and ligation without arterial reconstruction, excision with immediate reconstruction, and excision with interval reconstruction.

## Introduction

Mycotic aortic aneurysm (MAA) patients have a serious pathology that can be fatal if not diagnosed early and managed efficiently [1]. The term mycotic was derived from the appearance of fresh fungus vegetation [2]. Despite its name, different organisms can cause infection of the aorta, such as bacteria in most cases, followed by fungi. This infection leads to the destruction of the aortic endothelium, causing aneurysmal dilatation, which may rupture in some cases [3]. MAAs can develop as a result of direct bacterial inoculation into the arterial wall, bacteremic seeding of existing intimal damage, atherosclerotic plaque, aneurysm, contiguous infection, or septic emboli. Diagnostic difficulties based on history and physical findings should be followed by laboratory and imaging techniques (computed tomography (CT) with contrast and angiography, magnetic resonance angiography, digital subtraction angiography, and blood cultures). Local expansion of a thoracic MAA can elicit compressive symptoms such as dysphagia, dyspnea, hoarseness, cough, and superior vena cava syndrome; however, if diagnosed late, thoracic MAA can cause devastating complications such as rupture or bleeding.

The microbiology of mycotic aneurysms has changed in recent years due to antibiotic evolution. In the pre-antibiotic era, infection was mainly secondary to infective endocarditis causative organisms such as *Streptococcus *(including *Streptococcus pneumonia*), *Staphylococcus*, *Haemophilus *spp., and *Pneumococcus*, and inoculation was usually in the aortic arch rather than other aortic parts [4]. However, this has changed due to the widespread use of antibiotics, which decreased endocarditis frequency and shifted inoculation incidence to the side of *Staphylococci and Salmonella *spp., with more affection of the abdominal aorta [5].

*Salmonella *spp. is a common cause of infection in humans, which can result in serious complications such as mycotic aneurysms. We report this interesting and challenging case with a rare presentation, which is one of the rare reports of a patient with mycotic thoracic aortic aneurysm caused by *Salmonella enterica* infection.

## Case presentation

A 74-year-old Saudi female with diabetes mellitus and hypertension presented to the emergency department with fever, abdominal pain, mild dysuria, and generalized fatigue lasting 10 days. She had a COVID-19 infection seven months ago and was on 2 L of oxygen through a nasal cannula. The patient was admitted to the Buraidah Central Hospital’s Internal Medicine Department with a fever of 39°C, suspected of having COVID-19 or a urinary tract infection (UTI). The initial laboratory results showed raised white blood cells (WBCs), predominantly neutrophils, aberrant C-reactive protein (CRP), high erythrocyte sedimentation rate (ESR) and lactate dehydrogenase, and normal renal and hepatic function tests.

After admission, a COVID-19 swab was negative, and a workup was performed, including a CT of the abdomen and pelvis, which revealed bilateral lung bases with atelectatic changes and advanced atherosclerotic changes of the abdominal aorta and iliac arteries. The patient was provisionally diagnosed with acute bronchitis due to cough and shortness of breath associated with a UTI and was given medications in addition to oxygen. A blood culture was requested, and the results were positive for *Salmonella enterica* serotype *typhi *(*S. typhi*), which was biochemically identified and serologically confirmed. After five days of daily ceftriaxone therapy, the patient was discharged from the hospital due to a decrease in fever, dysuria, abdominal pain, and overall stability.

After 10 days, she returned to the emergency department with a complaint of diarrhea for five days and intermittent fever and chills for three days, which were accompanied by shortness of breath and abdominal pain that did not improve with analgesics. The patient was examined and found to be tachypneic (26 beats/minute) and febrile (39.4°C), with a regular pulse rate of 120 beats/minute and blood pressure of 135/80 mmHg. There were crepitations in the right scapula and decreased air entry in the left scapula. The abdomen was mildly distended but soft. There were no other notable findings in the general examination. Venous blood gases revealed hypoxemia, necessitating 5 L of nasal cannula oxygen to maintain saturation at 94% (pH = 7.53, PaCO_2_ = 30.4, PaO_2_ = 55, HCO_3_ = 30.4, O_2_ saturation = 93%). The hemogram showed leukocytosis at 13 × 10^3^/µL (reference range = 4.5-11 × 10^3^/µL), with 70% neutrophilia (reference range = 37-80%) and a normal platelet count of 190 × 10^3^/µL (reference range = 150-450 × 10^3^/µL). The urine examination, renal function tests, and liver function tests were all normal.

The patient was admitted again with a suspected UTI rather than hospital-acquired pneumonia. Fever was intermittent and partially responded to antipyretics, with increased WBCs (mainly neutrophilic). WBC was 23.6 × 10^3^/µL, neutrophil was 10.6 × 10^3^/µL, and hemoglobin was 8.8 g/dL (reference range = 12-16 g/dL). The urine analysis showed an increased WBC count. The acute-phase reactants showed an ESR of 120 mm/hour (reference range = 0-20 mm/hour) and a CRP level of 239 mg/L (reference range = <5 mg/L). A chest X-ray revealed right lower lobe consolidation. Microbiology pan-culture was done (sputum culture, urine culture, and blood culture). The urine culture was positive for *Escherichia coli*. The sputum culture was negative. *S. enterica* was detected again in the blood culture (unexplained persistent *S.* *enterica *bacteremia). Empirical piperacillin/tazobactam was given for three days with no improvement in symptoms, so meropenem was added to basal, HR insulin, and prophylactic anticoagulant.

**Figure 1:**
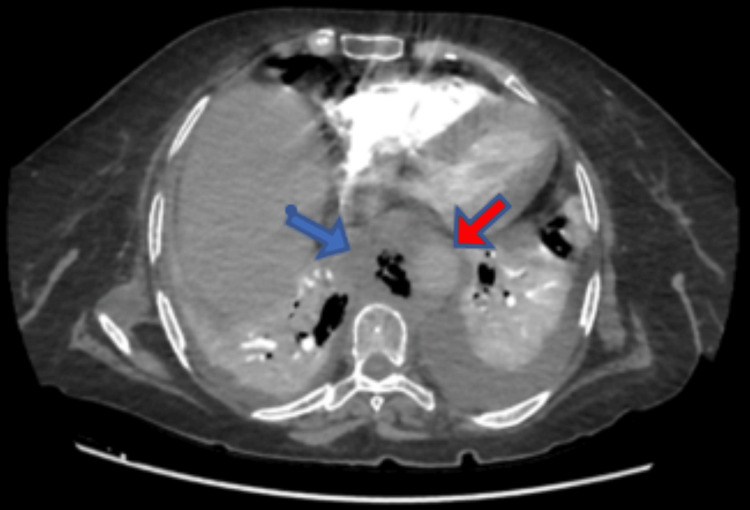
Mycotic aneurysm (blue arrow) and aorta (red arrow).

On day four of admission, a high-resolution CT of the chest was requested to confirm the pneumonia diagnosis and its extent. CT revealed cardiomegaly, coronary calcification, and bilateral mild pleural effusion with passive subsegmental collapse, no pneumothorax, and large anterior paraspinal mixed soft tissue with air density displacing the thoracic aorta anteriorly with an impression of air within the aortic wall. Hence, a contrast study during the venous and arterial phases was performed immediately to further clarify the previous radiologic finding, which revealed a saccular aortic aneurysm on the right side of the lower thoracic descending aorta measuring 1.7 × 2.5 cm with an adjacent abscess formation measuring 5.5 × 6 × 6.7 cm. The results were consistent with an aortic mycotic aneurysm with an adjacent abscess (Figure 1).

The immediate vascular surgery team was notified and requested that the patient be transferred to a higher-level center in Riyadh for urgent intervention.

The patient was transferred as a life-saving case to King Fahad Medical City in Riyadh, where urgent thoracic endovascular aortic repair (TEVAR) with stenting was performed, and meropenem was administered via a peripherally inserted central catheter. Unfortunately, the patient experienced left lower limb deep vein thrombosis and pulmonary embolism, necessitating the use of a therapeutic anticoagulant (low-molecular-weight heparin), which was later switched to a new oral anticoagulant with intermittent pneumonic compression.

The patient gradually stabilized on meropenem through the peripherally inserted central catheter for at least six weeks tailored to culture and sensitivity. The patient improved in terms of previous complaints and general condition. The patient displayed no distress, lying comfortably in bed with no active chest pain or abdominal pain, normal vital signs, and sustained saturation of 1-2 L, which improved to room air later. The patient remained in the hospital for approximately 40 days after TEVAR with intravenous antibiotics, with follow-up of acute-phase reactants, cultures, and sensitivity, as well as overall health.

## Discussion

It is difficult to suspect a case of MAA because of the absence of specific symptoms and the unapproachability of screening tests. Hence, MAAs include *Salmonella *mycotic aortic aneurysm (SMAA) in the differential diagnosis workup as early assessment can prevent catastrophic complications that can decrease morbidity and mortality [6].

SMAAs are uncommon in the thoracic aorta but can occur in immunocompromised patients with one or more of the following comorbidities: hypertension, diabetes, and atherosclerosis [7], which was the case with our patient. However, it can also increase in those with acquired immunodeficiency syndrome, chronic kidney disease, or autoimmune diseases treated with biological agents [7]. *S. enterica* infection is rarely serious enough to cause complications, except in those with low immunity [8]; however, most patients experience a variety of symptoms, ranging from mild gastroenteritis, abdominal pain, and fever that do not require extensive treatment to bacteremia, which can result in a poor outcome and death [9].

Vascular intima is resistant to infection by its nature, but it can be easily jeopardized by different risk factors that prepare it for a bacterial invasion that spreads to deeper layers leading to suppuration, pseudoaneurysm formation, or even perforation [10]. Because thoracic MAA is uncommon compared to other MAAs, few studies have been conducted to document its natural history. It is unclear if its pattern follows the other thoracic aortic aneurysms [11].

In Asian countries, *S. typhimurium*, *S. enteritidis*, and *S. choleraesuis* are the most commonly reported pathogens for thoracic MAA bacterial inoculation, while *S. aureus*, *Salmonella *spp., and *Pseudomonas aeruginosa* are more prevalent in Western countries [12].

Thoracic MAA is uncommon, accounting for approximately 30% of MAAs affecting other parts of the aorta, with a male-to-female incidence ratio of 3:1, which may be due to higher atherosclerotic aortic disease prevalence in males. This incidence is more prevalent at the age of 65-70 years, which corresponds to our patient’s age; however, our patient was female rather than male [13].

In terms of presenting symptoms in thoracic MAA, according to the literature, few patients are asymptomatic with incidental discovery of the aneurysm. However, in the majority of patients, fever, chest and back pain, abdominal pain, and chills are the main findings in MAA (resembling the non-specific symptoms of MAA) [14,15], and the majority of these symptoms were recorded in our patient.

Diagnosis of thoracic MAA is difficult without a suspicion of its presence, and as there are no specific screening tests, TMAA should be considered a differential diagnosis in risky patients [6].

Laboratory findings, such as CRP, leukocytosis, and ESR, are usually higher than normal in recorded cases [16], in addition to positive blood cultures for either staphylococci, non-typhoidal *Salmonella*, or streptococci, though some were found to be negative [17,18].

However, contrast-enhanced CT and angiographic imaging of the aorta are the most important diagnostic tools, as they also influence management plans and surgical timing [19]. In our patient, the main diagnosis was a positive blood culture for *S. enterica*, followed by contrast-enhanced CT on the arterial and venous phases, which confirmed the diagnosis.

Successful management of MAA depends on early diagnosis and effective treatment based on rapid, aggressive surgical debridement and occasional reconstruction with either open or endovascular procedures, which constitute the first and effective treatment step, along with an appropriate and complete antibiotics course [20].

## Conclusions

Thoracic MAA is a rare condition with high morbidity and mortality rates. Gram-negative bacteria, particularly *S. enteridis*, are more commonly found as causative organisms in people with comorbidities. As seen in our patient, the symptoms were non-specific and vague, making early thoracic MAA diagnosis a true challenge. Efficient treatment requires a combination of surgical repair with pre- and post-surgery antibiotic use. Thus, thoracic MAA is an important diagnosis to consider in elderly patients with symptoms that point to an undefinable state of infection.
